# Loading dose vitamin D_3_ improves vitamin D insufficiency in adults undergoing hematopoietic stem cell transplantation: A randomized controlled trial

**DOI:** 10.1371/journal.pone.0284644

**Published:** 2023-10-26

**Authors:** Ni Bai, Karen Lee, Wasithep Limvorapitak, Emily Liu, David Kendler, Raewyn Broady, Jennifer White

**Affiliations:** 1 Division of Hematology, Department of Medicine, Leukemia Bone Marrow Transplant Program of British Columbia, University of British Columbia, Vancouver, Canada; 2 Division of Hematology, Department of Internal Medicine, Faculty of Medicine, Thammasat University, Rangsit Campus, Pathumthani, Thailand; 3 Division of Endocrinology, Department of Medicine, University of British Columbia, Vancouver, Canada; University of Malaya Faculty of Medicine, MALAYSIA

## Abstract

Allogeneic hematopoietic stem cell transplant (aHSCT) patients are well known to be at high risk of vitamin D (vit D) deficiency. This study assessed whether a loading dose (100,000 IU) of vitamin D_3_ pre-aHSCT could effectively achieve and maintain sufficient post-transplant vit D levels (serum total 25 hydroxy vitamin D (25(OH)D) ≥ 75nmol/L). Dual-energy X-ray absorptiometry (DXA) was also conducted for bone health evaluation. 74 patients were enrolled and randomly assigned, in a 1:1 ratio, either to the high vit D group (single loading dose (100,000 IU) plus 2,000 IU vit D_3_ daily) or the control group (2,000 IU vit D_3_ daily). Vit D levels were measured at three time points (baseline, day 30 and day 100 post-aHSCT). At baseline, fewer than 50% patients had a sufficient 25(OH)D (control: 42.9%; high vit D: 43.6%). The proportion of patients with sufficient 25(OH)D (nmol/L) was increased at day 30 and day 100, with a trend of higher proportion in the high vit D group at day 30 (high vit D vs. control: 89.7% vs. 74.3%, p = 0.08). The increased 25(OH)D was significantly higher in the high vit D group at day 30 (high vit D vs. control: 29±25.2 vs. 14 ±21.9, p = 0.01). Insufficient vit D level before transplant (baseline) was an independent risk factor for vit D insufficiency (serum 25(OH)D < 75nmol/L) post-aHSCT (OR = 4.16, p = 0.03). DXA suggested significant bone loss for total hip in both groups, and in the femoral neck for the control group only. In conclusion, single loading dose vitamin D_3_ significantly increased total 25(OH)D levels at day 30 post-transplant, and the intervention was especially beneficial for patients with baseline vit D insufficiency. We acknowledge that the primary outcome at day 100 post-aHSCT indicating superiority of loading dose versus daily dose supplementation was not met.

## Introduction

Allogeneic hematopoietic stem cell transplant (aHSCT) patients are well known to be at high risk of vitamin D (vit D) deficiency both pre- and post-transplant [1–3]. The prevalence of vit D insufficiency (serum total 25 hydroxy vitamin D (25(OH)D) < 75nmol/L) is reported to be as high as 70% before and 90% after transplant [1–4]. Vit D plays an essential role in maintaining bone health as well as extraskeletal benefits. Vit D insufficiency leads to decreases in bone mineral density (BMD), which can lead to osteopenia or osteoporosis, with consequent increases in fracture risk [5, 6]. Rates of osteopenia up to 50% and osteoporosis up to 30% of aHSCT patients have been reported [7–11].

In addition to the essential role of vit D in maintaining bone health, vit D also exerts potent immunomodulatory effects [12]. By binding and activating vit D receptors that are expressed in several cell types in the immune system, including in T lymphocytes, dendritic cells and natural killer cells [13]. Vit D downregulates pro-inflammatory immune cells and subsequently their cytokine production, while enhancing the anti-inflammatory subsets, thus mediating inflammation and fostering a more tolerogenic environment [14]. Vit D insufficiency has been linked to various autoimmune disorders, including systemic lupus erythematosus [15, 16], multiple sclerosis, inflammatory bowel disease [17], and rheumatoid arthritis [18]. In the context of aHSCT, an association between vit D insufficiency and graft versus host disease (GVHD) has been reported in adult aHSCT patients [1]. Patients who underwent aHSCT and developed acute GVHD at 100 days post-transplant were associated with lower vit D levels, and pre-transplant vit D deficiency was reported to have higher risk and/or greater severity chronic GVHD [19–21]. In one prospective multicenter trial, investigators found that vit D supplementation up to 100 days post-transplant significantly attenuated the risk of chronic GVHD at year one post-aHSCT [22].

Similar to a report in the pediatric literature [23], we conducted a retrospective chart review of 89 patients and revealed that at 100 days post-aHSCT, 47% aHSCT recipients were vit D insufficient despite having been prescribed 2,000 IU vit D_3_ daily [24]. These results suggest that transplant patients may require higher doses of vit D than the general population due to transplant related risk factors, including high dose conditioning, chemotherapy and radiation, reduced mobility, and the immunosuppressive therapies [1, 7, 9, 11, 25]. A study conducted in pediatric aHSCT patients (mean age 5.8 ± 4.9 years) showed that administrating a single, ultra-high dose of vit D (maximum 600,000 IU) before transplant was able to maintain sufficient 25 hydroxy vitamin D levels for at least 8 weeks [26]. However, no studies have been conducted in the adult aHSCT population. Following American Society for blood and Marrow Transplantation management guidelines [27], we advised all patients undergoing aHSCT to take 2,000 IU vit D_3_ daily. However, the recommended regimens were not able to efficiently correct vit D insufficiency for aHSCT recipients [24]. A recent survey conducted in 114 centers from 24 European countries indicated that evidence-based guideline to manage vit D insufficiency in patients undergoing aHSCT is warrant, especially the potential role of vit D in post-transplant complications [13].

100,000 IU vit D_3_ monthly dosing is the equivalent of 4,000 IU vit D_3_ daily, and has been considered as the tolerable upper limit from the Endocrine Society clinical practice guideline [27]. It has been reported that an oral dose of 100,000 IU vit D_3_ was safe and effective in maintaining sufficient vit D levels in individuals at high risk for vit D insufficiency [26, 28, 29]. Thus, the objective of this study was to assess whether a 100,000 IU loading dose of vit D_3_ prior to aHSCT, followed by 2000 IU daily, could effectively achieve and maintain sufficient vit D levels post-transplant for aHSCT patients.

## Patients and methods

### Study population

Patients, age 18 or above, at the Leukemia/Bone Marrow Transplant Program of British Columbia undergoing aHSCT during May 2018 to June 2019 were screened and invited to participate in the study. Individuals with a history of urolithiasis, hypercalcemia, hypervitaminosis D (25(OH)D >375 nmol/L), or sensitivity to vit D supplements were excluded. There was no vit D level requirement or restriction to be enrolled to this study. The study protocol was reviewed and approved by the University of British Columbia Research Ethics Board. All enrolled patients provided written informed consent. This study was registered at ClinicalTrials.gov (NCT03534674).

### Randomization and intervention

Eligible patients were randomly assigned by the research team, in a 1:1 ratio, either to the control group or the high vit D (intervention) group on the day of admission for aHSCT using permuted blocks of four with stratification by conditioning regimen (myeloablative vs. reduced intensity). Participants were not informed of the intervention assignment. Patients in the control group received our standard 2,000 IU (JAMP Pharma, CA) vit D_3_ daily. Those who were assigned to the high vit D group received a single oral loading dose of 100,000 IU vit D_3_ (JAMP Pharma, CA) on the second day of hospital admission, then continued with 2,000 IU vit D_3_ daily. Vit D supplementation was administered in liquid form or through total parenteral nutrition for patients who were unable to take oral medications.

### Data collection

One of the most reliable markers of vit D status is serum 25 hydroxy vitamin D (25(OH)D). In the general population, vit D insufficiency and deficiency were defined as the concentrations of 25(OH)D between 21 to 29 ng/mL (53 to 73 nmol/L), or less than 20 ng/mL (50 nmol/L) respectively [30, 31]. Whereas some studies used 50 nmol/L as a threshold to define vit D deficiency [2, 22, 32]. In this study, vit D sufficiency was defined as 25(OH)D ≥75nmol/L, below which was deemed as vit D insufficient, which includes both insufficiency and deficiency to accommodate the inconsistency of different cut-offs used by other studies [33].

Serum 25(OH)D levels were measured by liquid chromatography-tandem mass spectroscopy (SCIEX API 5000 Triple Quad Mass Spectrometer) at three time points: pre-aHSCT (baseline), day 30 and day 100 post-aHSCT. The magnitude of change in 25(OH)D for each patient, and the proportion of patients with normal 25(OH)D at post-aHSCT were then calculated, respectively. The total amount of vit D_3_ given for each patient during the inpatient stay was obtained by chart review, and assumed 2,000 IU daily vit D_3_ subsequent to hospital discharge as this was the recommendation for each patient.

To evaluate the potential contribution of vit D to post-transplant complications, we assessed the effects of serum 25(OH)D on bone health using dual-energy x-ray absorptiometry (DXA) at pre-aHSCT (baseline) and day 100 post-aHSCT, and categorized using the World Health Organization definition: normal: T-score > –1.0 SD, osteopenia: T-score between -1.0 and –2.5 SD, osteoporosis: T-score ≤ –2.5 SD [21]. GVHD was diagnosed and graded according to standard criteria [22]. The incidence of acute GVHD and chronic GVHD was analysed at day 100 and year one post-aHSCT, respectively. Acute GVHD was diagnosed and scored according to Glucksberg criteria. Severity of chronic GVHD was assessed according to the National Institutes of Health (NIH) criteria [34]. All patients’ demographics and transplant-related information were obtained from their medical records.

### Statistical analysis

Sample size was derived from the primary objective, based on results of our previous study showing that the average serum 25(OH)D levels were 50 nmol/L at baseline and increased to 72 nmol/L at day 100 post-aHSCT [24]. The standard deviation of the mean change in 25(OH)D from baseline to day 100 post-aHSCT was 18.6 among 89 patients (61%) who had vit D insufficiency. In order to detect a 25% additive increase (from 61% to 86%) in the proportion of patients achieving sufficient serum 25(OH)D level (≥ 75 nmol/L) at day 100 post-aHSCT, we needed 38 subjects in each group. We would therefore be able to reject the null hypothesis that the mean change in 25(OH)D levels between the vit D group and control group were equal with 80% power and a type I error of 5%, using one-sided t-test. To account for a maximum 10% transplant-related mortality at day100 post-transplant, we planned to enroll 84 patients (42 in each group).

Our analysis followed a modified intention-to-treat principle. Participants who died or relapsed between the time of randomization and day 100 post-aHSCT were excluded from the analysis as these participants lacked day 100 25(OH)D results. We did not use statistical techniques to impute missing data (eg, by multiple imputation), because we judged that the missing data were scarcity (eg. less than 5%).

Two sample *t* test was conducted for between group comparisons. Chi-square test was performed to compare the proportion of patients achieving sufficient serum 25(OH)D. Paired *t* test was used to compare BMD T-scores within each group before and after the transplant. Spearman analysis was conducted for association analyses. To examine the impact of potential confounding factors (e.g. age, sex, disease, transplant type), linear regression models were employed with post-transplant vit D levels as the dependent variable. Univariate models were performed and factors with a univariate p<0.20 were included into multivariable model. These analyses were performed using Stata software version 13.0 (StataCorp LP, Texas, USA).

## Results

### Patient characteristics

**Fig 1:**
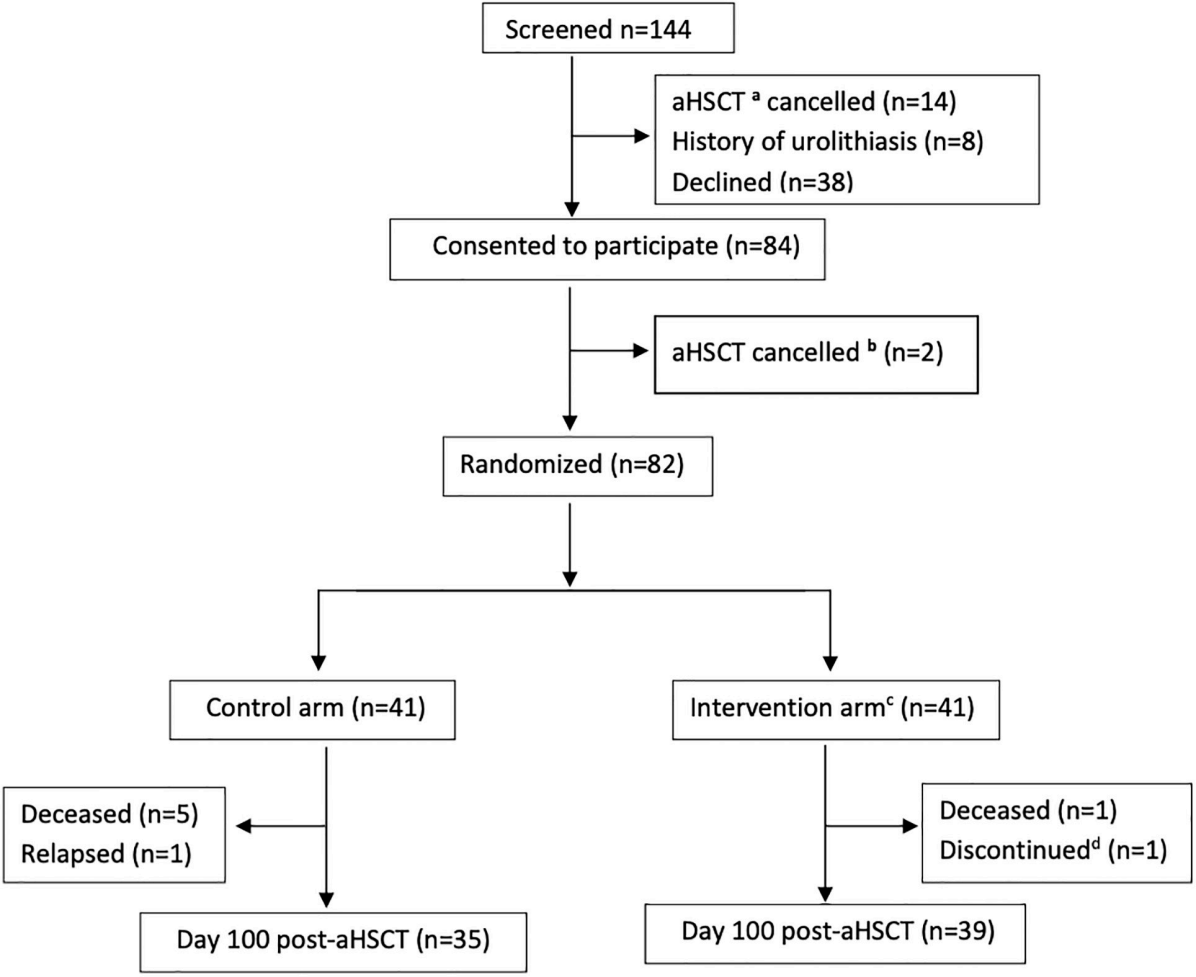
CONSORT diagram. Study population and randomization profile. a: aHSCT: Allogeneic hematopoietic stem cell transplantation; b: The transplant was cancelled in 14 patients prior to the randomization; c: High vit D group (single oral loading dose of 100,000 IU vit D_3_); d: One patient was taken off the study due to a medical condition unrelated to the study intervention 25(OH)D levels and the effect of loading vit D_3_.

144 patients were screened, and a total of 74 patients were included in the modified intent-to-treat analysis (Fig 1). The baseline characteristics of patients are described in Table 1. The vit D group was significantly older (high vit D vs. control (years): 55±13.3 vs. 46±16.1, p = 0.01), and had a relatively higher proportion of female subjects (high vit D vs. control: 16±41% vs. 10±28.6%, p = 0.26). The majority of patients had acute leukemia (high vit D: 20 (51%); control 24 (69%)) and underwent myeloablative transplantation (high vit D: 32 (82.1%); control 27 (77.1%)). Independent variables (sex, age, disease diagnosis, and type of transplant) were not deemed to be significant risk factors for insufficient 25(OH)D at day 100 post-aHSCT (data in S1 Table). The average hospital admission days were similar for patients in control (35 days) and the high vit D group (36 days). Except for the loading dose vit D in the high vit D group, patients in two groups had no statistical difference for cumulative vit D supplementation taken during hospitalization within 100 days of post-transplant (data in S2 Table).

**Table 1:** Patient and transplant characteristics of modified intention-to-treat population.

Patient Characteristic	Control (n = 35)	High Vit D (n = 39)	P-value
Age at transplant (years)			
Mean (SD)	46 (16.1)	55 (13.3)	0.01*
Median (Range)	45 (19–69)	59 (21–67)	0.04*
Sex			0.26
Male (N, %)	25 (71.4)	23 (59.0)	
Female (N, %)	10 (28.6)	16 (41.0)	
Diagnosis			0.28
ALL	10	5	
AML	14	15	
CLL	1	1	
CML	2	0	
MDS	2	0	
MYF	1	5	
NHL	3	4	
RAEB	1	5	
Others	1	4	
Type of transplant			0.60
Myeloblative (N, %)	27 (77.1)	32 (82.1)	
RIC (N, %)	8 (22.9)	7 (17.9)	
Acute GVHD at 100-day post-aHSCT with overall grade II-IV	14 (40.0)	14 (35.9)	0.65
Chronic GVHD at one-year post-aHSCT with moderate to severe	9 (25.7)	12 (30.8)	0.16

ALL: acute lymphoblastic leukemia; AML: acute myeloid leukemia; NHL: non-Hodgkin lymphoma; CLL: chronic lymphocytic leukemia; MDS: myelodysplastic syndrome; MYF: myelofibrosis; RAEB: refractory anemia with excess blasts; RIC: reduced intensity conditioning; SD: standard deviation.

*P value<0.05.

**Fig 2:**
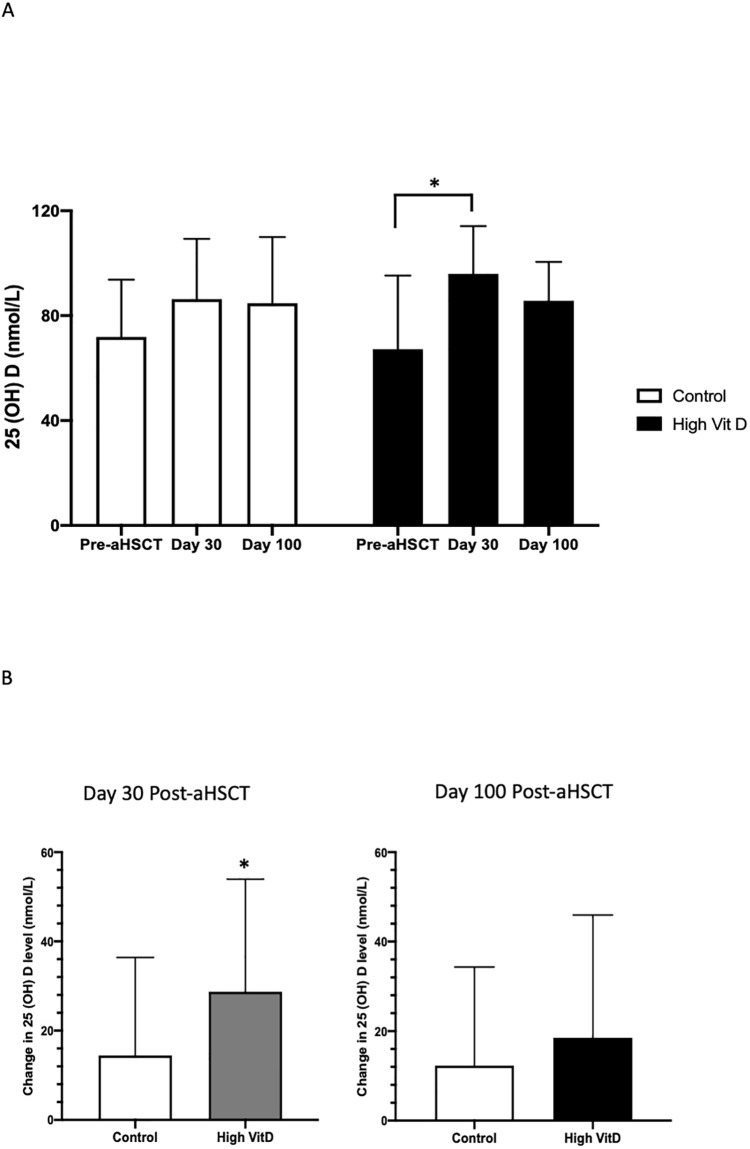
25(OH)D levels pre- and post-aHSCT by study groups. (A) 25(OH)D levels increased in both groups at day 30 and day 100 post-aHSCT. The increase was significantly greater in the high Vit D group at day 30; however, this significant elevation was no longer apparent at day 100 post-aHSCT; (B) The magnitude of change in 25(OH)D level at day 30 post-aHSCT was significantly higher in the vit D group, compared with the control. However, this difference was no longer apparent at day 100 post-aHSCT. *P <0.05.

Before transplant, mean 25(OH)D levels did not differ between two groups (high Vit D vs. Control (nmol/L): 67±28.1 vs. 71 ±21.9, p = 0.42) (Table 2). 25(OH)D increased in both groups at day 30 and day 100 post-aHSCT (Fig 2 & Table 2). Patients in the intervention group had a significantly greater increase in 25(OH)D at day 30 post-aHSCT (high Vit D vs. control (nmol/L): 29±25.2 vs. 14±21.9, p = 0.01); however, this increase was not sustained at day 100 (high Vit D vs. control (nmol/L): 19±27.4 vs. 12±22.0, p = 0.29) (Fig 2). Nevertheless, the increase at day 100 was still greater in the vit D group compared to the control group (Table 2).

**Table 2:** 25 (OH)D levels (nmol/L) pre- and post-aHSCT.

	Control (n = 35)	High Vit D (n = 39)	P value
Pre-aHSCT			
Mean (SD)	71 (21.9)	67 (28.1)	0.42
Median (range)	70 (40–150)	68 (15–120)	
Day 30 post-aHSCT			
Mean (SD)	86 (22.9)	96 (18.2)	0.05
Median (range)	90 (35–146)	95 (57–154)	
Day 100 post-aHSCT			
Mean (SD)	83 (28.0)	86 (14.8)	0.55
Median (range)	82 (34–169)	88 (50–112)	
The proportion of patients with normal 25(OH)D (≥75nmol/L)			
Pre-aHSCT (%, N)	42.9% (15)	43.6% (17)	0.95
Day 30 post-aHSCT (%, N)	74.3% (26)	89.7% (35)	0.08
Day 100 post-aHSCT (%, N)	65.7% (23)	82.1% (32)	0.11

aHSCT: Allogeneic hematopoietic stem cell transplant; SD: standard deviation.

At baseline, fewer than 50% patients had a normal 25(OH)D (control group: 42.9%; high Vit D supplement group: 43.6%) (Table 2). Compared with pre-transplant, the proportion of patients with normal 25(OH)D in both groups was increased at day 30 and day 100 post-aHSCT, with a trend to a greater proportion having normal 25(OH)D in the vit D group at day 30 (high Vit D vs. control: 89.7% vs. 74.3%, p = 0.08), and this trend continued at day 100 (high Vit D vs. control: 82.1% vs. 65.7%, p = 0.11) (Table 2). Notably, 82.1% of patients in the vit D group sustained normal 25(OH)D levels from day 30 to day 100, compared to 65.7% of patients in the control group (Table 2).

**Fig 3:**
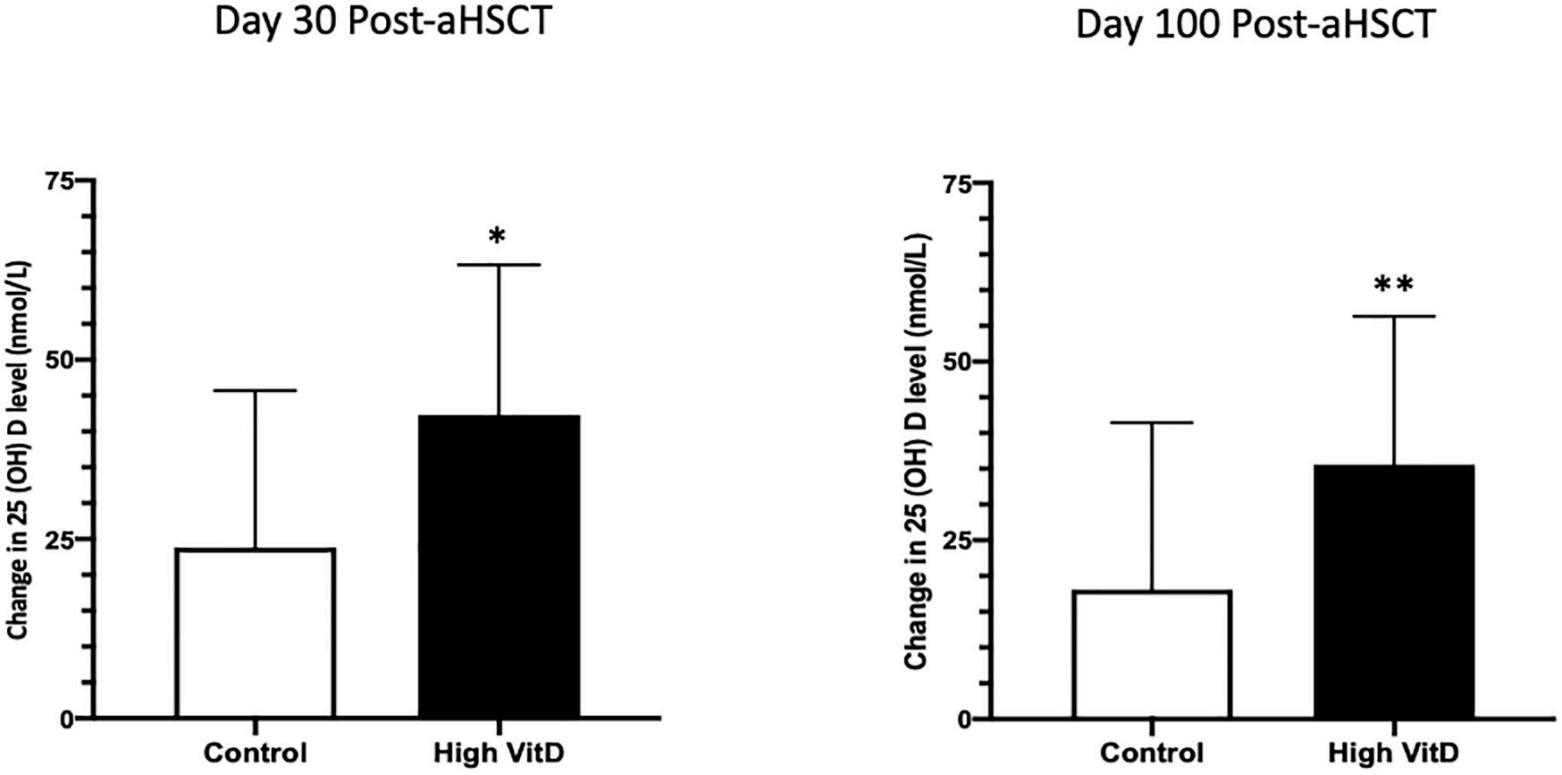
The magnitude changes of 25(OH)D levels in subgroup that was comprised of patients with insufficient 25(OH)D levels at baseline (pre-aHSCT). Comparing with the control, the magnitude change was significantly increased in the intervention group at both day 30 and day 100 post-aHSCT. *P = 0.008, **P = 0.01.

Univariable and multivariate regression analyses revealed that sex, age, disease diagnosis, or type of transplant were not associated with low 25(OH)D (Data in S1 Table). However, insufficient baseline 25(OH)D (<75nmol/L) was a risk factor for vit D insufficiency post-aHSCT (OR = 3.89, 95% CI = 1.14–13.21, p = 0.03). Therefore, we conducted additional subgroup analysis for patients with insufficient baseline 25(OH)D. Although subgroup analysis did not reveal statistically significant improvement in serum 25 (OH) D after high dose supplements (Table 3), the magnitude of increase in serum 25(OH)D in this subgroup was significantly higher for patients in the vit D group, at both day 30 post-aHSCT (high Vit D vs. Control (nmol/L): 42±21.0 vs. 24 ±21.9, p = 0.008), and day 100 post-aHSCT (high Vit D vs. control (nmol/L): 36±20.8 vs. 18 ±23.3, p = 0.01) (Fig 3) (Table 3). The subgroup analysis also indicated that the increase of proportion patients in the intervention group was greater at day 30 or day 100 post-aHSCT compared to the control group (Table 3).

**Table 3:** Subgroup 25(OH)D levels (nmol/L) and the proportion of patients with normal 25(OH)D at post-transplant.

	Control (n = 20)	High Vit D (n = 22)	P value
25(OH) D levels			
Pre-aHSCT			
Mean (SD)	58 (11.3)	47 (17.7)	0.01*
Median (range)	57 (40–72)	47 (15–74)	
Day 30 post-aHSCT			
Mean (SD)	80 (25.0)	90 (15.2)	0.16
Median (range)	85 (35–146)	94 (57–116)	
Day 100 post-aHSCT			
Mean (SD)	77 (20.6)	86 (14.8)	0.32
Median (range)	76 (34–115)	88 (15–120)	
The proportion of patients with normal vit D			
Day 30 post-aHSCT	65%	82%	0.22
Day 100 post-aHSCT	50%	77%	0.07

25(OH)D: 25 hydroxy vitamin D; aHSCT: Allogeneic hematopoietic stem cell transplant;

SD: standard deviation.

*P <0.05.

### Bone mineral density (BMD)

Both baseline and day 100 BMD data were available for 27 patients (77%) in the control group and 32 patients (82%) in the intervention group. After transplant, BMD at lumbar spine, total hip, and femoral neck were all within the normal range (≥ -1.0), but BMD declined significantly at total hip in both groups at day 100. Femoral neck BMD was significantly lower after transplant in the control group (pre vs. post-aHSCT: -0.4±1.6 vs. -0.7±1.7, p = 0.04) (Table 4). Lumbar spine BMD did not significantly differ pre- and post-transplant. Overall, there was a trend of decline in BMD and increasing osteopenia in both groups post-transplant (Table 4). Associations between pre-aHSCT or post-aHSCT vit D levels and BMD were not identified, respectively (Spearman correlations, p>0.05) (data in S1 Fig).

**Table 4:** T-scores for bone mineral density (BMD) test.

	Control (n = 27)			High Vit D (n = 32)		
	Pre-aHSCT	100-day post-aHSCT		Pre-aHSCT	100-day post-aHSCT	
T-score			P value^2^			P value^2^
**Total Hip**						
Mean (SD)	-0.2 (1.4)	-0.6 (1.5)	0.0003*	-0.3 (1.0)	-0.6 (0.9)	0.01*
Median (range)	-0.4 (-2.6- +3.2)	-0.8 (-1.3- +3.8)		-0.2 (-2.1- +1.8)	-0.5 (-2.5- +1.0)	
Normal BMD^1^ (N, %)	17 (63%)	14 (52%)		24 (75%)	20 (63%)	
Osteopenia^1^ (N, %)	8 (30%)	10 (37%)		8 (25%)	11 (34%)	
Osteoporosis^1^ (N, %)	2 (7%)	3 (11%)		0 (0%)	1 (3%)	
**Femoral Neck**						
Mean (SD)	-0.4 (1.6)	-0.7 (1.7)	0.04*	-0.7 (1.1)	-0.9 (1.0)	0.13
Median (range)	-0.8 (-2.6- +3.4)	-1.2 (-1.4- +4.2)		-0.8 (-2.5- +1.9)	-0.9 (-2.7- +0.7)	
Normal BMD (N, %)	15 (56%)	11 (42%)		16 (52%)	15 (48%)	
Osteopenia (N, %)	9 (33%)	13 (50%)		14 (45%)	15 (48%)	
Osteoporosis (N, %)	3 (11%)	2 (8%)		1 (3%)	1 (4%)	
**Lumbar Spine**						
Mean (SD)	-0.5 (1.5)	-0.5 (1.6)	0.79	-0.3 (1.4)	-0.5 (1.5)	0.40
Median (range)	-0.7 (-3.2-+3.8)	-0.7 (-2.3- +4.9)		-0.5 (-2.3- +3.3)	-1.05 (-2.5-+2.7)	
Normal BMD (N, %)	16 (59%)	14 (54%)		18 (56%)	14 (44%)	
Osteopenia (N, %)	10 (37%)	11 (42%)		14 (44%)	17 (53%)	
Osteoporosis (N, %)	1 (4%)	1 (4%)		0 (0%)	1 (3%)	

BMD: Bone mineral density; SD: standard deviation.

*P <0.05.

### Acute and chronic GVHD

By day 100 post-aHSCT, 14 (40.0%) patients in the control and 14 (35.9%) in the intervention group developed acute GVHD with overall grade II-IV. The cumulative incidence and overall grade were not significantly different between groups (Table 1). 8 (23%) patients in the control or intervention group had gut GVHD, and no association between vit D insufficiency and gut GVHD was identified. By 1-year post-aHSCT, the incidence for chronic GVHD (moderate to severe) was not significantly different between groups (Table 1). A correlation between vit D insufficiency and the incidence of GVHD was not revealed (Data in S3 to S6 Tables).

## Discussion

To the best of our knowledge, this study is the first randomized controlled trial investigating the effectiveness of a loading dose vit D (100,000 IU) in improving post-aHSCT 25 (OH) D levels in adult aHSCT recipients. Our findings provide important information on vit D supplementation regimens for this population who are at high risk of vit D deficiency.

The prevalence of vitD insufficiency in our study cohort is similar to previous reports demonstrating insufficient vit D before transplant [1, 2, 7, 35]. Our study demonstrates that there were clear early benefits of loading vit D_3_ in these patients. We found that at day 30 and day 100 post-transplant, serum 25 (OH) D levels, the increase in 25 (OH)D, and the proportion of patients with normal 25(OH)D, were all significantly higher from baseline (Tables 2 and 3 & Figs 2 and 3). The improvements in 25(OH)D peaked at day 30 post-aHSCT, indicating a positive early effect of vit D_3_ loading (Figs 2 and 3). The finding of a decline of 25(OH)D levels at day 100 was consistent with 25(OH)D pharmacokinetics characterized previously by Ilahi et al [36] who showed the time course of serum 25(OH)D after administration of single dose of 100,000 IU vit D_3_ in a healthy population. Given that transplantation, including high dose conditioning, chemotherapy and radiation and the immunosuppressive therapies, are associated with vit D insufficiency [1, 7, 9, 11, 26], this effect attenuated day 100 post-aHSCT, perhaps due to the improved health status of these patients later post-transplant, but a more thorough discussion of this issue was beyond the scope of this manuscript. The more rapid achievement of vit D sufficiency at day 30 post-aHSCT may suggest a potential benefit of repeated post-aHSCT loading or high dose strategy in aHSCT patients. A pitfall of the study was the number of patients with vit D insufficiency was lower than what we observed in the previous study that was used to calculate the sample size; hence the sample size in this study may not be powered as expected.

Patients with baseline insufficient vit D_3_ seemed to show the greatest benefit of loading dose supplementation (Fig 3 & Table 3). Evidence from other studies, including the pediatric population, also showed that the effectiveness of aggressive vit D supplementation could prevent vit D insufficiency early after aHSCT [20, 23, 26]. A study conducted in pediatric aHSCT patients (mean age 5.8 ± 4.9 years) demonstrated that administrating a single, ultra-high dose of vit D (maximum 600,000 IU) before the transplant was able to maintain normal vit D levels to 8 weeks [26]. Their investigation showed serum 25(OH)D peaked at 7 days (median), subsequently fell back to baseline in 70 days. Findings from our investigation and from previous studies would suggest that future trials should determine 25(OH) D levels early post-transplant, such as measuring vit D levels at 30-day post-aHSCT. This may be the time at which a loading dose of high vit D would be beneficial to maintain sufficient vit D levels. Alternatively, a higher daily dose of vit D_3_ such as 4000 IU daily in this at-risk population may be considered to ensure vit D sufficiency. A particular focus on patients with inadequate pre-transplant 25(OH) D is warranted since this is a strong predictor of insufficient vit D levels after transplant. The study loading dose of 100,000 IU vitamin D_3_ was chosen arbitrarily as the upper limit of normal cumulative daily dose from the Institute of Medicine recommendations. Although a higher dose of vit D may have led to a more rapid achievement of vit D sufficiency, our study did not have the scope to investigate a range of vit D dosing.

A positive correlation between low post-transplant 25(OH) D and low BMD was previously demonstrated [9]. We found that BMD was normal in the majority of pre- and post-transplant patients. More than 50% of patients had normal BMD at baseline and the proportion of patients with normal BMD did not significantly change after transplant (Table 4). These results contrast data from our previous study which revealed a significant decline in femoral neck BMD, and increased frequency of both osteopenia and osteoporosis at day 100 post-transplant [10, 37]. The low prevalence of osteoporosis in the present study cohort may have impaired our ability to detect bone loss in both groups by day 100 [9]. In addition, as a standard of care in our transplant program, zoledronic acid infusion was given to eight patients in both groups due to low baseline BMD (T-score<-1.5), rendering any positive effects of vit D supplementation difficult to detect. Although the BMD T-scores in both groups were within the normal range, total hip and femoral neck experienced greater declines compared to the lumbar spine post-aHSCT, similar to what we previously reported [10]. Schulte and Beelen [9] also demonstrated that rapid early decline of BMD was more pronounced at the femoral neck compared to spine during the first-year post-transplant, followed by progressive bone loss for the total observation period of 4 years. The reasons for different rates of bone loss at different skeletal sites following aHSCT remain unclear.

It has been reported that bone loss was greatest in the first 6 months post-transplant [38], then progressive over for 2–6 years [9, 39]. We did not observe a significant increase in osteopenia or osteoporosis, though the day 100 time point may be too early to appreciate BMD differences. To prevent the progression to osteoporosis in aHSCT recipients, serial BMD scans may be imperative to identify and manage osteoporosis at an early stage [7, 8]. In addition, prospective studies with longer follow-up are warranted to evaluate long-term impact of vit D status on bone loss.

There is increasing interest in the immunomodulatory effects of vit D in the context of aHSCT [12, 14]. Vit D deficiency in aHSCT recipients has been associated with an increased incidence of GVHD, and patients suffering from acute GVHD (III-IV) have been reported to have low vit D levels [19, 21]. However, whether vit D supplementation would prevent or ameliorate the course of GVHD remains a question [12, 14]. We did not identify significant differences in the incidence or severity of acute or chronic GVHD post-aHSCT (Table 1). Our study was admittedly not powered to investigate the effect of vit D supplementation on GVHD, though these relationships would be an interesting area for future investigations, especially for patients with low baseline vit D. Moreover, whether low 25(OH) D is associated with GVHD pathophysiology or whether improved 25(OH)D can contribute to the development of tolerance in patients and prevention of GVHD remains to be clarified and addressed in future prospective studies.

In summary, this study showed for the first time that administration of a single loading dose vit D was safe and well-tolerated in adult aHSCT recipients, and resulted in significantly higher vit D levels at day 30 post-transplant, compared with the control who received standard vit D supplementation. The benefit was most apparent among patients with vit D insufficiency at pre-transplant baseline who had significantly greater increases at both day 30 and day 100 post-aHSCT. Therefore, measuring pre-transplant vit D is recommended, and aggressive vit D supplementation for patients with low baseline vit D may prevent post-aHSCT vit D insufficiency. Future investigations with frequent vit D measures early post-transplant may offer valuable insights regarding loading dose vit D either pre-transplant or early post-transplant. In addition, longer-term prospective studies may help to shed light on the role of vit D supplementation in preventing bone loss post-aHSCT.

## Supporting information

S1 Fig The association between vit D levels and BMD. (DOCX)

S1 Table Univariable regression analysis for risk factors associated with insufficient vit D levels on day 100 post-aHSCT. (DOCX)

S2 Table Comparison of vit D intake from aHSCT to day 100 post-aHSCT. (DOCX)

S3 Table The association between baseline vit D levels and acute GVHD (aGVHD). (DOCX)

S4 Table The association between insufficient vit D levels at D100 and acute GVHD (aGVHD). (DOCX)

S5 Table The association between baseline vit D levels and chronic GVHD (cGVHD). (DOCX)

S6 Table The association between D100 vit D levels and chronic GVHD (cGVHD). (DOCX)

S1 Checklist Reporting checklist for randomised trial. (PDF)

S1 File (DOCX)

S1 Data (XLSX)
