# Supplementary material for: Loading dose vitamin D3 improves vitamin D insufficiency in adults undergoing hematopoietic stem cell transplantation: A randomized controlled trial
Source: PLoS One. 2023 Oct 26;18(10):e0284644. doi: 10.1371/journal.pone.0284644 (PMC10602320; doi:10.1371/journal.pone.0284644)
Supplement: S1 Table — (DOCX) [file pone.0284644.s002.docx]

S1 Table. Univariable regression analysis for risk factors associated with insufficient vit D levels on day 100 post-aHSCT.

| Factors | Odds ratio (OR) | 95% confidence interval | P-value |
| --- | --- | --- | --- |
| Age (for every 10-year increase) | 0.66 | 0.47 – 0.93 | 0.019 |
| Female sex | 0.58 | 0.18 – 1.84 | 0.353 |
| Diagnosis group*  - Acute myeloid leukemia and  Myelodysplastic syndrome  - Acute lymphoblastic leukemia  - Other | 1  1.56  1.78 | Reference  0.45 – 5.35  0.42 – 7.50 | 0.483  0.434 |
| Reduced intensity conditioning | 0.16 | 0.02 – 1.33 | 0.020 |
| Pre-aHSCT insufficient vit D levels | 3.89 | 1.14 – 13.21 | 0.030 |
| High dose vit D given | 0.42 | 0.14 – 1.23 | 0.113 |

*Diagnosis of non-Hodgkin lymphoma was dropped as there was only one patient with

insufficient vit D at day 100 post-aHST.
 **P-value < 0.2 was included in multivariable regression analysis.
