# Supplementary material for: Loading dose vitamin D3 improves vitamin D insufficiency in adults undergoing hematopoietic stem cell transplantation: A randomized controlled trial
Source: PLoS One. 2023 Oct 26;18(10):e0284644. doi: 10.1371/journal.pone.0284644 (PMC10602320; doi:10.1371/journal.pone.0284644)
Supplement: S2 Table — (DOCX) [file pone.0284644.s003.docx]

S2 Table. Comparison of vit D intake from hospital admission for aHSCT to day 100 post-aHST.

|  | Control | High Vit D | P-value |
| --- | --- | --- | --- |
| Vit D intake (IU)  (Excluding standard does in the control and loading dose in the high Vit D group) | 193,446 | 190,994 | 0.8 |
