# Supplementary material for: Loading dose vitamin D3 improves vitamin D insufficiency in adults undergoing hematopoietic stem cell transplantation: A randomized controlled trial
Source: PLoS One. 2023 Oct 26;18(10):e0284644. doi: 10.1371/journal.pone.0284644 (PMC10602320; doi:10.1371/journal.pone.0284644)
Supplement: S3 Table — (DOCX) [file pone.0284644.s004.docx]

S3 Table. The association between baseline vit D levels and acute GVHD (aGVHD).

|  | aGVHD | non-aGVHD | P value |
| --- | --- | --- | --- |
| Vit D < 75 nmol/L | 16 | 26 | 0.6 |
| Vit D ≥ 75 nmol/L | 14 | 18 |  |
