# Supplementary material for: Loading dose vitamin D3 improves vitamin D insufficiency in adults undergoing hematopoietic stem cell transplantation: A randomized controlled trial
Source: PLoS One. 2023 Oct 26;18(10):e0284644. doi: 10.1371/journal.pone.0284644 (PMC10602320; doi:10.1371/journal.pone.0284644)
Supplement: S4 Table — (DOCX) [file pone.0284644.s005.docx]

S4 Table. The association between insufficient vit D levels at D100

and acute GVHD (aGVHD).

|  | aGVHD | non-aGVHD | P value |
| --- | --- | --- | --- |
| Control | 9 | 3 | 0.2 |
| High vit D | 3 | 4 |  |
