# Supplementary material for: Loading dose vitamin D3 improves vitamin D insufficiency in adults undergoing hematopoietic stem cell transplantation: A randomized controlled trial
Source: PLoS One. 2023 Oct 26;18(10):e0284644. doi: 10.1371/journal.pone.0284644 (PMC10602320; doi:10.1371/journal.pone.0284644)
Supplement: S5 Table — (DOCX) [file pone.0284644.s006.docx]

S5 Table. The association between baseline vit D levels and chronic GVHD (cGVHD).

|  | cGVHD | non-cGVHD | P value |
| --- | --- | --- | --- |
| Vit D < 75 nmol/L | 29 | 13 | 0.3 |
| Vit D ≥ 75 nmol/L | 18 | 14 |  |
