# Supplementary material for: Loading dose vitamin D3 improves vitamin D insufficiency in adults undergoing hematopoietic stem cell transplantation: A randomized controlled trial
Source: PLoS One. 2023 Oct 26;18(10):e0284644. doi: 10.1371/journal.pone.0284644 (PMC10602320; doi:10.1371/journal.pone.0284644)
Supplement: S6 Table — (DOCX) [file pone.0284644.s007.docx]

S6 Table. The association between D100 vit D levels and chronic GVHD (cGVHD).

|  | cGVHD | non-cGVHD | P value |
| --- | --- | --- | --- |
| Vit D < 75 nmol/L | 15 | 7 | 0.7 |
| Vit D ≥ 75 nmol/L | 35 | 20 |  |
