# Supplementary material for: Loading dose vitamin D3 improves vitamin D insufficiency in adults undergoing hematopoietic stem cell transplantation: A randomized controlled trial
Source: PLoS One. 2023 Oct 26;18(10):e0284644. doi: 10.1371/journal.pone.0284644 (PMC10602320; doi:10.1371/journal.pone.0284644)
Supplement: S1 File — (DOCX) [file pone.0284644.s009.docx]

**LEUKEMIA/BONE MARROW TRANSPLANT PROGRAM OF BRITISH COLUMBIA**

**Division of Hematology**

| Division of Hematology  2775 Laurel Street, 10^th^ Floor  Vancouver, BC V5Z 1M9  Phone: 604.875.4863  Fax: 604.875.4763  [www.leukemiabmtprogram.com](http://www.leukemiabmtprogram.com) |
| --- |
| Supported by:  BC Cancer Agency  Vancouver General Hospital  University of British Columbia |
| *Director:*  Raewyn Broady  MBChB, FRACP, FRCPA |
| *Members:*  Yasser R. Abou Mourad  MD, FRCPC  Michael J. Barnett  BM, FRCPC, FRCP, FRCPath  Donna L. Forrest  MD, FRCPC  Alina S. Gerrie  MD, MPH, FRCPC  Donna E. Hogge  MD, PhD, FRCPC  Stephen H. Nantel  MD, FRCPC  Sujaatha Narayanan  MBBS, MRCP, FRCPath  Thomas J. Nevill  MD, FRCPC  Maryse M. Power  MB, MRCPI, FRCPath  Kevin W. Song  MD, FRCPC  Heather J. Sutherland  MD, PhD, FRCPC  Cynthia L. Toze  MD, MHSc, FRCPC |

**Validating a Vitamin D3 Supplementation Regimen**

**among Patients Undergoing Allogeneic**

**Hematopoietic Stem Cell Transplantation**

**Current version date:** February 15, 2019

**Current version**: 2.0

**Principal Investigator:**

Raewyn Broady, MBChB, FRACP, FRCPC

Leukemia/Bone Marrow Transplant Program of BC

Division of Hematology, Department of Medicine

University of British Columbia

British Columbia Cancer Agency

Phone: (604) 875-4863

Email: RBroady@bccancer.bc.ca

**Co-Investigator:**

David Kendler, MD, FRCPC

Division of Endocrinology

University of British Columbia

Phone: (604) 263-3661

**Project Coordinator**

Nicola Bai

Leukemia/Bone Marrow Transplant Program of BC

Division of Hematology, Department of Medicine

University of British Columbia

British Columbia Cancer Agency

Phone: (604) 875-4111, ext: 69013

Email: nicola.bai@bccancer.bc.ca

**TABLE OF CONTENTS**

1. BACKGROUND [3](#_Toc495001633)

[1.1 Allogeneic Hematopoietic Stem Cell Transplantation and Bone Deterioration 3](#_Toc495001638)

[1.2 Vitamin D Deficiency among aHSCT Patients 3](#_Toc495001640)

[1.3 Vitamin D and Immunomodulatory Funciton 4](#_Toc495001640)

2. [STUDY RATIONALE 4](#_Toc495001634)

3. [OBJECTIVES 5](#_Toc495001634)

[4. STUDY DESIGN 5](#_Toc495001637)

[4.1 Study Randomization 5](#_Toc495001638)

[4.2 Arm A (Control Group) 5](#_Toc495001639)

[4.3 Arm B (Intervention Group) 5](#_Toc495001640)

[4.4 Data Collection and Endpoints 5](#_Toc495001639)

[4.5 Baseline Demographics 5](#_Toc495001639)

[5. Eligibility and Study Entry Criteria 6](#_Toc495001637)

[5.1 Inclusion Criteria 6](#_Toc495001638)

[5.2 Exclusion Criteria 6](#_Toc495001639)

[5.3 Informed Consent 6](#_Toc495001640)

[5.4 Protocol Approval 7](#_Toc495001640)

[6. STUDY PROCEDURE 7](#_Toc495001642)

[6.1 Informed Consent 7](#_Toc495001638)

[6.2 Confirmation of Eligibility 7](#_Toc495001639)

[6.3 Baseline Demographics 7](#_Toc495001640)

[6.4 Biochemisity and Bone Mineral Density Analysis 7](#_Toc495001640)

[6.5 Immune and inflammatory biomarkers 8](#_Toc495001640)

[7. SAFETY PROCEDURES 8](#_Toc495001648)

[8. STATISTICAL METHODS 8](#_Toc495001652)

[8.1 Randomization 8](#_Toc495001653)

[8.2 Determination of Sample Size 8](#_Toc495001654)

[8.3 Analyses Supporting the Primary Objective 9](#_Toc495001655)

[8.4 Analyses Supporting Secondary Objectives 9](#_Toc495001656)

[9. REFERENCES 11](#_Toc495001652)

[10. FIGURES 12](#_Toc495001652)

[10.1 Figure 1 14](#_Toc495001653)

[10.2 Figure 2 15](#_Toc495001654)

#

1. **BACKGROUND**

- 1. *Allogeneic Hematopoietic Stem Cell Transplantation and Bone Deterioration*

Allogeneic hematopoietic stem cell transplantation (aHSCT) is a potentially curative therapy for many patients with hematological malignancies. aHSCT is however associated with significant morbidity that includes severe loss of bone mineral density (BMD), a key determinant of future risk of fractures. A 10-15% decrease in BMD approximately doubles the risk of fracture (1), which can negatively influence quality of life and lead to premature death for long-term aHSCT survivors (2).

Transplant-related BMD loss is a rapidly evolving, and long-lasting disorder. The loss of BMD following aHSCT can be attributable to multiple factors, such as high-dose chemotherapy, the use of immunosuppressive therapy, and decreased activity (3). BMD is significantly lower in pre-aHSCT population than in age-matched general population (4). Further bone loss can occur as early as 100 days post-transplant (5) and progress over for 2-6 years, leading to persistent osteoporosis (6-8) and higher fracture rates (3).

*1.2 Vitamin D Deficiency* *among aHSCT Patients*

Vitamin D, as the precursor to the potent steroid hormone calcitriol, plays an important role in regulation of the systemic calcium, and is essential to preserve bone mass. Vitamin D deficiency can be resulted from the lack of sun exposure, impairment of liver and kidney function, and chronic use of corticosteroids (9-11). Vitamin D deficiency may also play a role in bone loss, but has not been carefully studied to date in the aHSCT population.

Osteoporosis, caused by a loss of bone tissue, can be measured by dual energy X ray absorptiometry *(*DXA), which is one of the most accurate and precise methods for measuring bone mineral density and determine the risk for fractures.

In a review of 265 patients undergoing aHSCT at the Leukemia/BMT Program of British Columbia during Jan, 2012 to July, 2016, we found that 62% patients were vitamin D insufficient or deficient at the baseline (pre-aHSCT) (Figure1). It has been reported by others that 60-70% aHSCT survivors have vitamin D deficiency (8,11). Vitamin D supplementation has been strongly recommended for aHSCT patients (12-14); however, 47% of patients failed to achieve sufficient 25-OH-D3 levels by 100 days post-aHSCT by taking recommended regimen, 2000 IU vitamin D3 daily (Figure 2).

*1.3 Vitamin D and Immunomodulatory Function*

Immunomodulatory actions of vitamin D have been recognized for over a few decades (15,16), but it is only in the last few years that the significance of this to normal human physiology. It has become apparent after a number of observations have suggested that vitamin D is a key factor linking innate and adaptive immunity, and both of these functions may be compromised under conditions of vitamin D insufficiency (17-20).

Vitamin D binds and activates vitamin D receptor, which is expressed in several cell types in the immune system, including in T lymphocytes and antigen presenting cells, such as dendritic cells (10;11). Vitamin D has been implicated in promoting the production and function of T regulatory cells which are critical mediators of immune system (10). Vitamin D can also act directly on T lymphocytes to inhibit their proliferation (12).  Studies have shown that vitamin D supplementation promotes better immune function in the reconstituted immune system (21, 22).

**2. STUDY RATIONALE**

Sustained optimal vitamin D level is essential in preventing bone loss after aHSCT. However, studies from us (Figure 2) as well as others have shown that an optimal 25-OH-D3 level is difficult to achieve in aHSCT recipients by a vitamin D3 regimen recommending 2000 IU vitamin D3 daily (23).

Studies have demonstrated that an oral loading dose of 100,000 IU vitamin D3 is safe and helpful in maintaining an adequate 25-OH-D3 level in people with various medical conditions, including patients who were critically ill (24-26).

Significant bone loss initiates before aHSCT and progresses rapidly at the early stage of post-aHSCT, therefore, prophylactic interventions can be essential to prevent severe bone loss and osteoporosis in long-term survivors of aHSCT. We propose a clinical trial randomizing patients to standard of care which is vitamin D3 2000 IU per day versus 100,000 IU vitamin D loading dose with 2000 IU per day.

The primary endpoint would be to achieve and maintain sufficient 25-OH-D3 levels (>75 nmol/L) in patients 100 days after undergoing aHSCT at the Leukemia/BMT Program of British Columbia.

**3. OBJECTIVES**

*3.1 Primary Objective*

To assess the efficacy (the proportion of patients achieving sufficient serum 25-OH-D3 level) after a single oral loading dose of 100,000 IU vitamin D3 prior to aHSCT, with subsequent vitamin D3 2000 IU daily, in preventing vitamin D insufficiency/deficiency in the first 100 days post-aHSCT.

*3.2 Secondary Objective*

To compare the effect of a single oral 100,000 IU vitamin D3 loading dose with our standard of care (2000 IU vitamin D3 daily) in terms of

1. BMD of spine and hip, trabecular bone score (TBS) at three months and one year post aHSCT.

2. Immunological and inflammatory biomarkers.

**4. STUDY DESIGN**

*4.1 Study Randomization*

This study will randomize patients 1:1 using block randomization.

*4.2 Arm A (Control Group)*

Participants assigned to the control group will be advised to take our current vitamin D regimen (2000 IU vitamin D3 daily).

*4.3 Arm B (Intervention Group)*

Participants assigned to the intervention group will receive a single oral loading dose of 100,000 IU vitamin D3 on the second day of hospital admission for aHSCT, then 2000 IU vitamin D3 daily.

The vitamin D3 2000 IU daily supplement will be provided on discharge for all study participants.

*4.4 Data Collection and Endpoints*

Serum 25-OH-D3 levels will be measured at baseline (pre-aHSCT), 30 and 100 days post-aHSCT. As vitamin D is involved in the regulation of serum phosphate and calcium, we will also measure serum calcium, phosphate, ALP, and PTH at these time points. To monitor bone health, bone mineral density (BMD) will be assessed by dual-energy x-ray absorptiometry (DXA) at baseline, 100 days and one year post-aHSCT as standard of care.

*4.5 Baseline Demographics*

Demographic data will be collected by patient interviews at baseline, including age, other health conditions, fracture risk factors, calcium and vitamin D intake. Medical and transplantation variables will be obtained from patients' medical records.

# 5. Eligibility and Study Entry Criteria

Eligible patients will be identified by chart review prior to the transplant. Inclusion and exclusion criteria must be met as outlined in Sections 5.1 and 5.2 before the patients are randomized. Randomization will be done on the day of admission for aHSCT at the Leukemia/BMT Program of British Columbia.

##### 5.1 Inclusion Criteria

1. Patients undergoing allogeneic stem cell transplantation at the Leukemia/BMT Program of British Columbia.
2. Age greater than or equal to 18 years.
3. Able to provide written informed consent.

##### Exclusion Criteria

1. History of kidney stones, hypercalcemia, hypervitaminosis D, or allergic/ sensitive to vitamin D.

2. Not meeting eligibility criteria to proceed with allogeneic stem cell

transplantation as per the Leukemia/BMT Program of BC.

##### 5.3 Informed Consent

The participants must acknowledge in writing their consent to be a study subject.

##### 5.4 Protocol Approval

This protocol must be reviewed and approved by the Research Ethics Board (REB) prior to approaching participants for this study.

# 6. STUDY PROCEDURES

Blood work will be sampled at baseline (pre-aHSCT), post-aHSCT day 30, and day 100. Bone mineral density (BMD) will be assessed by dual-energy x-ray absorptiometry (DXA) at baseline, 100 days and one year post-aHSCT, respectively, as part of standard of care.

*6.1 Informed Consent*

The subject must read, understand, and sign the REB-approved informed consent form (ICF) confirming his or her willingness to participate in this study. In addition, subjects must sign all approved ICF amendments per the REB guidelines during the course of the study.

*6.2 Confirmation of Eligibility*

All necessary procedures and evaluations will be performed to document that the subject meets all eligibility criterion (Section 5).

*6.3 Baseline Demographics*

Demographic data will be collected by patient interviews at baseline, including age, medical history and baseline medications. Medical and transplantation variables will be obtained from patients' medical records.

*6.4 Biochemistry and Bone Mineral Density Analysis*

Serum 25-OH-D3 levels will be measured pre-transplant (baseline), 30 and 100 days post-aHSCT, respectively. Measurements of baseline biochemistry (calcium, phosphate, ALP, PTH) will be conducted at pre-transplant, day 30, 100, and one year post-aHSCT. BMD by DXA scan will be performed at pre-transplant, day100, and one year post-aHSCT, respectively, as part of our routine standard of care.

*6.5 Immune and inflammatory biomarkers*

We will connect this study to Hematology Cell Bank of BC and the Legacy Cell Bank of BC in which blood samples are collected at the time of diagnosis and subsequent follow-up periods. This linkage will allow us to obtain study samples without additional phlebotomy. We will request in writing (email) to the project coordinator to obtain blood samples matching proposed study time points.

Markers of immune function including total white blood cell count, lymphocyte count, CD4+ and CD8+ subsets of T-lymphocytes, and T-regulatory cells will be tested at pre- and day 100 post-transplant. Studies from other (6a) as well as Dr. Broady's laboratory specialized in T-regulatory cell function in the aHSCT population have demonstrated the role of T-regulatory cells in the pathogenesis of GVHD (27, 28)

# 7. SAFETY PROCEDURE

# We do not anticipate adverse events attributable to this single dose vitamin D3 intervention.

**8. STATISTICAL METHODS**

##### 8.1 Randomization

Subjects will be randomized into either the control group or the intervention group using 1:1 block randomization.

##### 8.2 Determination of Sample Size

Sample size is derived from the primary objective, based on results of our previous study showing that the average serum 25-OH-D3 levels were 50nmol/L at baseline and increased to 72 nmol/L at 100 days post-HSCT. The standard deviation of the mean change in 25-OH-D3 from baseline to 100 days post-aHSCT was 18.6 among 89 patients (61%) who had vitamin D3 insufficiency. In order to detect a 25% additive increase (from 61% to 86%) in the proportion of patients achieving sufficient serum 25-OH-D3 level (>75nmol/L) at day 100 post-aHSCT, we need 38 subjects in each group. We would therefore be able to reject the null hypothesis that the mean change in 25-OH-D3 levels between the intervention and control groups is equal with 80% power and a type I error of 5%, using one-sided t-test. To account for a maximum 10% transplant-related mortality at day100 post-transplant, we plan to enroll 84 patients (42 in each group).

*8.3 Analyses Supporting the Primary Objective*

The primary objective is to assess effect of a single oral loading dose of 100,000 IU vitamin D3 followed by 2000 IU vitamin D3 daily in preventing vitamin D insufficiency in aHSCT recipients, compared to 2000 IU vitamin D3 daily as the standard regimen.

Subjects who die between the time of randomization and day 100 will not be included in the analysis.

The primary analysis will be unadjusted and consist of a 2-sample, 1-sided, 0.05-level, t-test comparing the proportion of patients achieving normal serum 25-OH-D3 from baseline to 100 days post-aHSCT in control and intervention groups. To corroborate this analysis, a multivariable Gaussian regression model will be fit to include adjustment by age, sex, and disease (acute leukemia vs others).

The analysis described above will be corroborated by a per-protocol analysis to include only those subjects who provided both a baseline and 100 post-transplant serum 25-OH-D3.

*8.4 Analyses Supporting Secondary Objectives*

Analyses similar to those described for the primary objective will be performed.

#

**9. REFERENCES**

1 Holick MF: Optimal vitamin D status for the prevention and treatment of osteoporosis. Drugs Aging 2007;24:1017-1029.

2 Pundole XN, Barbo AG, Lin H, Champlin RE, Lu H: Increased incidence of fractures in recipients of hematopoietic stem-cell transplantation. J Clin Oncol 2015;33:1364-1370.

3 Robinson N, Sullivan KM. Complications of allogeneic bone marrow transplantation. Curr Opin Hematol. 1994 Nov;1(6):406-11. Review.

4 Välimäki MJ, Kinnunen K, Volin L, Tähtelä R, Löyttyniemi E,

Laitinen K, Mäkelä P, Keto P, Ruutu T. A prospective study of bone loss and turnover after allogeneic bone marrow tansplantation: effect of calcium supplementation with or without calcium. Bone Marrow Transplant. 1999; 23:355-361

5 Pawlowska M, Yang Q, Hamata B, Kendler DL, Broady R: Early changes in bone mineral density and trabecular bone score following allogeneic stem cell transplant. Bone Marrow Transplant 2016;51:738-740.

6 Abou-Mourad YR, Lau BC, Barnett MJ, Forrest DL, Hogge D Nantel SH, Shepherd JD, Smith CA, Song KW, Sitherland HJ, Toze CL, Lavoie JC. Long-term outcome after allo-SCT: close follow-up on a large cohort treated with myeloablative regimens. Bone Marrow Transplant 2010;45:295-320.

7 Kashyap A et al. Effects of allogeneic bone marrow

transplantation on receipient bone mineral desity: a prospective

study. Biol Blood Marrow Transplant. 2000; 6:344-351

8 Schulte CM, Beelen DW: Bone loss following hematopoietic stem cell transplantation: a long-term follow-up. Blood 2004;103:3635-3643.

9 Joseph, R.W.; Alousi, A.; Konda, B.; Komanduri, K.; Neumann, J; Trevino, C.; Stolar, K.; Qazilbash, M.; Hosing, C.; Kebriaei, P. et al, High incidence of vitamin D deficiency in patients undergoing allogeneic stem cell transplantation. *Am. J. Hematol.* **2011**, *86*, 954–956.

10 Robien, K.; Strayer, L.G.; Majhail, N.; Lazovich, D.; Baker, K.S.; Smith, A.R.; Mulrooney, D.A.; Burns, L.J. Vitamin D status among long-term survivors of hematopoietic cell transplantation. *Bone Marrow Transplant.* **2011**, *46*, 1472–1479.

11 Sproat L, Bolwell B, Rybicki L, Dean R, Sobecks R, Pohlman B, Andresen S, Sweetenham J, Copelan E, Kalaycio M: Vitamin D level after allogeneic hematopoietic stem cell transplant. Biol Blood Marrow Transplant 2011;17:1079-1083.

12 Dignan FL, Scarisbrick JJ, Cornish J, Clark A, Amrolia P, Jackson G, Mahendra P, Taylor PC, Shah P, Lightman S, Fortune F, Kibbler C, Andreyev J, Albanese A, Hadzic N, Potter MN, Shaw BE: Organ-specific management and supportive care in chronic graft-versus-host disease. Br J Haematol 2012;158:62-78.

13 Majhail NS, Rizzo JD, Lee SJ, Aljurf M, Atsuta Y, Bonfim C, Burns LJ, Chaudhri N, Davies S, Okamoto S, Seber A, Socie G, Szer J, Van Lint MT, Wingard JR, Tichelli A: Recommended screening and preventive practices for long-term survivors after hematopoietic cell transplantation. Hematol Oncol Stem Cell Ther 2012;5:1-30.

14 McClune BL, Polgreen LE, Burmeister LA, Blaes AH, Mulrooney DA, Burns LJ, Majhail NS: Screening, prevention and management of osteoporosis and bone loss in adult and pediatric hematopoietic cell transplant recipients. Bone Marrow Transplant 2011;46:1-9.

15 Lemire JM, Adams JS, Sakai R, Jordan SC. 1a,25-Dihydroxyvitamin D3

Suppresses Proliferation and Immunoglobulin Production by Normal Human Peripheral Blood Mononuclear Cells. J Clin Invest. 1984;74(August):657–61.

16. Provvedini M, Tsoukas CD, Deftos LJ, Manolagas SC. 1,25-

Dihydroxyvitamin D3 receptors in human leukocytes. Science.1983;80-:1181–3.

17. Rosen Y, Daich J, Soliman I, Brathwaite E, Shoenfeld Y. Scandinavian

Journal of Rheumatology Vitamin D and autoimmunity

Vitamin D and autoimmunity. Scand J Rheumatol. 2016;456:439–47.

18. Lopes Marques C. The Importance of vitamin D levels in autoimmune

disease. Bras. J Rheumatol. 2010;50:67–80.

19. Adorini L, Penna G. Control of autoimmune diseases by the

vitamin D endocrine system. Nat Clin Pract Rheumatol.2008;4:404–12.

20. Jose Ros-Soto ● Chloe Anthias, Alejandro Madrigal ● John A. Snowden. Vitamin D: is it important in haematopoietic stem cell transplantation? A review. Bone Marrow Transplantation

21. Silva F, Pérez-Simón JA, Caballero-Velazquez T, Sánchez-Guijo FM, Villanueva-Gomez F, Vazquez L, et al. . Effect of vitamin D treatment in chronic GVHD. Bone Marrow Transplant. (2011) 46:1395–7.

22. Caballero-Velázquez T, Montero I, Sánchez-Guijo F, Parody R, Saldaña R, Valcarcel D, et al. . Immunomodulatory effect of vitamin D after allogeneic stem cell transplantation: results of a prospective multicenter clinical trial. Clin Cancer Res. (2016) 22:5673–81. 10.1158/1078-0432.CCR-16-0238

23 Wallace G, Jodele S, Myers KC, Dandoy CE, El-Bietar J, Nelson A, Taggart CB, Daniels P, Lane A, Howell J, Teusink-Cross A, Davies SM: Vitamin D Deficiency in Pediatric Hematopoietic Stem Cell Transplantation Patients Despite Both Standard and Aggressive Supplementation. Biol Blood Marrow Transplant 2016;22:1271-1274.

24 Amrein K, Schnedl C, Holl A, Riedl R, Christopher KB, Pachler C, Urbanic PT, Waltensdorfer A, Munch A, Warnkross H, Stojakovic T, Bisping E, Toller W, Smolle KH, Berghold A, Pieber TR, Dobnig H: Effect of high-dose vitamin D3 on hospital length of stay in critically ill patients with vitamin D deficiency: the VITdAL-ICU randomized clinical trial. JAMA 2014;312:1520-1530.

25 Orgel E, Mueske NM, Sposto R, Gilsanz V, Wren TAL, Freyer DR, Butturini AM, Mittelman SD. A randomized controlled trial testing an adherence optimized Vitamin D regimen to mitigate bone change in adolescents being treated for acute lymphoblastic leukemia. Leuk Lymphoma. 2017; 58(10):2370-2378.

26 Wylon K, Drozdenko G, Krannich A, Heine G, Dolle S, Worm M: Pharmacokinetic Evaluation of a Single Intramuscular High Dose versus an Oral Long-Term Supplementation of Cholecalciferol. PLoS One 2017;12:e0169620.

27. Broady, R., Yu, J. & Levings, M. K. ATG-induced expression of FOXP3 in human CD4(+) T cells in vitro is associated with T-cell activation and not the induction of FOXP3(+) T regulatory cells. *Blood* **114,** 5003–6 (2009).

28. Broady, R. *et al.* Cutaneous GVHD is associated with the expansion of

tissue-localized Th1 and not Th17 cells. *Blood* **116,** 5748–51 (2010).

# 10. FIGURES

**38.11%**

**(101)**

**43.77%**

**(116)**

**17.36%**

**(116)**

N= 265

**Figure 1**

| Normal | >74 nmol/L |
| --- | --- |
| Mild deficiency/Insufficiency | 50-74 nmol/L |
| Moderate deficiency | 25-49 nmol/L |
| Severe deficiency | <25 nmol/L |

# Figure 2. Optimal vitamin D levels (>75nmol/L) were not achieved by standard vitamin regimen (2000 IU vitamin D3 daily) at 100 days post-transplant for patients with pre-transplant vitamin D insufficiency or deficiency (<75nmol/L).

#
